# Simultaneous Extraction of Density of States Width, Carrier Mobility and Injection Barriers in Organic Semiconductors

**DOI:** 10.1038/s41598-017-03882-8

**Published:** 2017-06-19

**Authors:** Pasquale Claudio Africa, Carlo de Falco, Francesco Maddalena, Mario Caironi, Dario Natali

**Affiliations:** 1 MOX Modeling and Scientific Computing, Dipartimento di Matematica, Politecnico di Milano, Piazza L. da Vinci 32, 20133 Milano, Italy; 2 Center for Nano Science and Technology @PoliMi, Istituto Italiano di Tecnologia, via Pascoli 70/3, 20133 Milano, Italy; 3 Dipartimento di Elettronica, Informazione e Bioingegneria, Politecnico di Milano, Piazza L. da Vinci 32, 20133 Milano, Italy

## Abstract

The predictive accuracy of state–of–the–art continuum models for charge transport in organic semiconductors is highly dependent on the accurate tuning of a set of parameters whose values cannot be effectively estimated either by direct measurements or by first principles. Fitting the complete set of model parameters at once to experimental data requires to set up extremely complex multi–objective optimization problems whose solution is, on the one hand, overwhelmingly computationally expensive and, on the other, it provides no guarantee of the physical soundness of the value obtained for each individual parameter. In the present study we present a step–by–step procedure that enables to determine the most relevant model parameters, namely the density of states width, the carrier mobility and the injection barrier height, by fitting experimental data from a sequence of relatively simple and inexpensive measurements to suitably devised numerical simulations. At each step of the proposed procedure only one parameter value is sought for, thus highly simplifying the numerical fitting and enhancing its robustness, reliability and accuracy. As a case study we consider a prototypical n-type organic polymer. A very satisfactory fitting of experimental measurements is obtained, and physically meaningful values for the aforementioned parameters are extracted.

## Introduction

Organic semiconductors are an outstanding candidate for becoming the material platform for the development of large–area, low cost, flexible electronics^1^. Since they can be processed from solution, they can be formulated as functional inks and deposited by means of printing techniques adapted from graphical arts (ink–jet, screen printing, spray coating, flexography to cite but a few)^2^. Their electronic performance has been constantly improving over the years leading to devices which compare well to, or even outperform, those based on amorphous silicon. Despite this technological progress, many fundamental questions are still debated and there is a strong need for simple yet reliable approaches to extract physical parameters from experimental measurements^3^.

Recently we have shown that, by fitting Capacitance-Voltage (CV) measurements of Metal–Insulator–Semiconductor (MIS) capacitors, it is possible to extract the width of the Density of States (DOS) – assuming it is a superposition of Gaussian functions – exploiting the sensitivity of CV curves to the semiconductor disorder degree^4, 5^. By operating MIS capacitors at suitably low frequency, quasi–equilibrium is ensured which implies that simulations can be performed in the static regime and that phenomena specifically related to carrier transport are negligible in order to fit experimental measurements, which leads to advantages in terms of both computational cost and accuracy. In addition, the DOS extraction is disentangled from carrier transport properties, which makes the fitting procedure substantially simpler and more robust.

**Figure 1:**
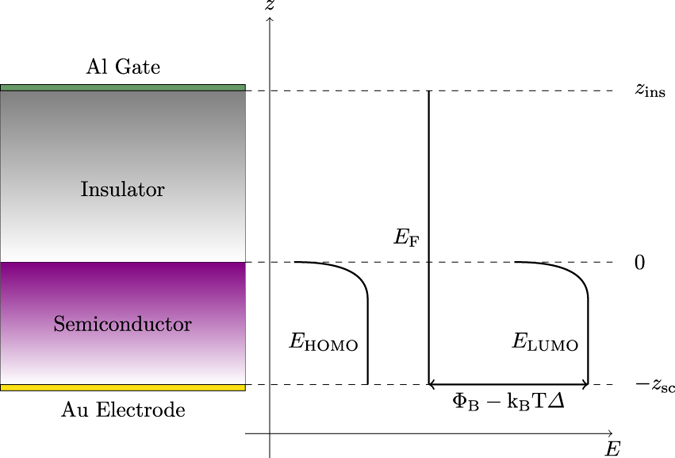
Schematic of the MIS capacitor used for the analysis and related energy levels.

If a DOS consisting of a single Gaussian provides a reasonable fit, the carrier mobility can be predicted in the framework of the Extended Gaussian Disorder Model (EGDM)^6^, and used to successfully fit the transfer characteristic curves of Organic Thin Film Transistors (OTFTs) in the linear regime. The schematic of the simulated/measured MIS capacitor is depicted in Fig. 1.

The extraction of the DOS width requires the accurate knowledge of the device geometrical dimensions, of the insulator and semiconductor permittivities, of the total density of available states and, most notably, of the metal/semiconductor injection barrier (Φ_B_) between the bottom metal and the semiconductor. The latter parameter is the one that suffers from the highest level of uncertainty: indeed, metal/semiconductor interfaces are still a subject of debate in the scientific community^7^; due to the various phenomena which may be involved (pillow effect, interface dipoles, charge transfer, chemisorption) the prediction of Φ_B_ is a hard task, and its measurement requires very dedicated equipment such as XPS/UPS^8^ or Kelvin Probe^9–11^.

**Figure 2:**
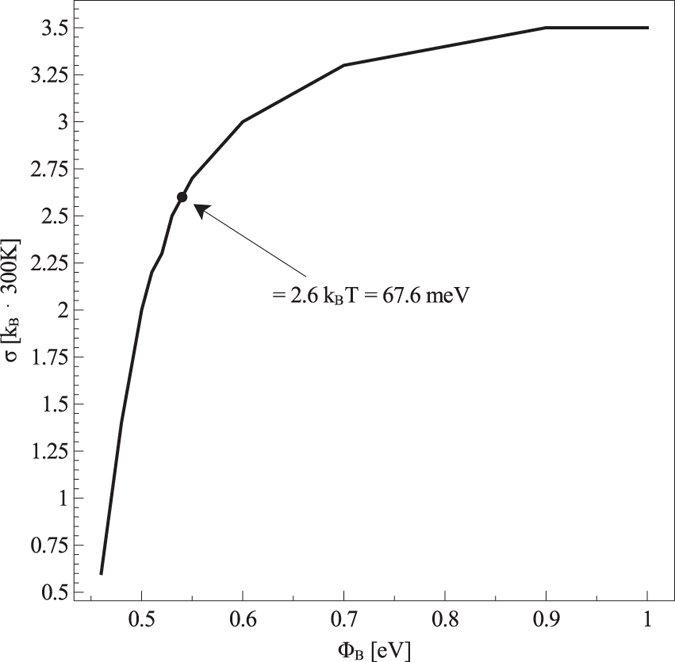
Dependence of the fitted Gaussian DOS width *σ* on the injection barrier Φ_B_. The dot on the curve identifies the *σ* value which simultaneously yields in the best fitting of OTFT transfer characteristic curves and of MIS capacitor CF curves. Experimental data are taken from ref. 4 and refer to MIS capacitors based on the prototypical n-type polymer P(NDI2OD-T2).

The uncertainty in Φ_B_ results in an uncertainty in determining the DOS width, as shown in ref. 4 and reported in Fig. 2: for each value of Φ_B_ a value for the DOS width can be fitted. Unfortunately, the fittings obtained by varying Φ_B_ are all of comparable quality. The uncertainty is not negligible indeed: by varying Φ_B_ from 1 eV down to 0.5 eV, the DOS width reduces from about 3.5 k_B_T down to about 0.5 k_B_T, which appears to be a rather unphysical value.

In the present work we demonstrate that this uncertainty can be drastically reduced by cooperatively exploiting MIS CV curves, MIS Capacitance–Frequency (CF) curves and OTFT transfer characteristic curves in the linear regime. To this end, we have extended our simulation domain so to cover out–of–equilibrium conditions in the framework of the Drift–Diffusion (DD) scheme. This enabled us to simulate the whole CF curve of the MIS capacitor. In addition, in the modeling and fitting of OTFT transfer characteristic curves we have taken into account the contact resistance in the context of the current crowding regime^12, 13^.

As a case study we choose as in ref. 4 Poly{[N,N′–bis(2-octyldodecyl)–naphthalene–1,4,5,8–bis(dicarboximide)–2,6–diyl]–alt–5,5′–(2,2′–bithiophene)} (P(NDI2OD-T2)), a printable, prototypical n-type polymer with a high mobility, exceeding 1 cm^2^ V^−1^ s^−1^ when processed from suitable pre–aggregating solvents^14, 15^. We find that the best fit to CV, CF and OTFT curves is obtained by assuming a Gaussian DOS width of 2.6 k_B_T and a barrier for electron injection from gold contacts of 0.54 eV. As to the former, the DOS width turns out to be slightly (13%) smaller than our former prediction^4^. As to the latter, Φ_B_ is considerably smaller than the nominal barrier, which is as large as 1 eV assuming that the Lowest Unoccupied Molecular Orbital (LUMO) lies at 4.0 eV and that the gold Fermi level (*E*_F_) lies at around 5 eV. The origin of such a small value for the barrier, which has already been postulated in other studies, is discussed.

## Results and Discussion

**Figure 3:**
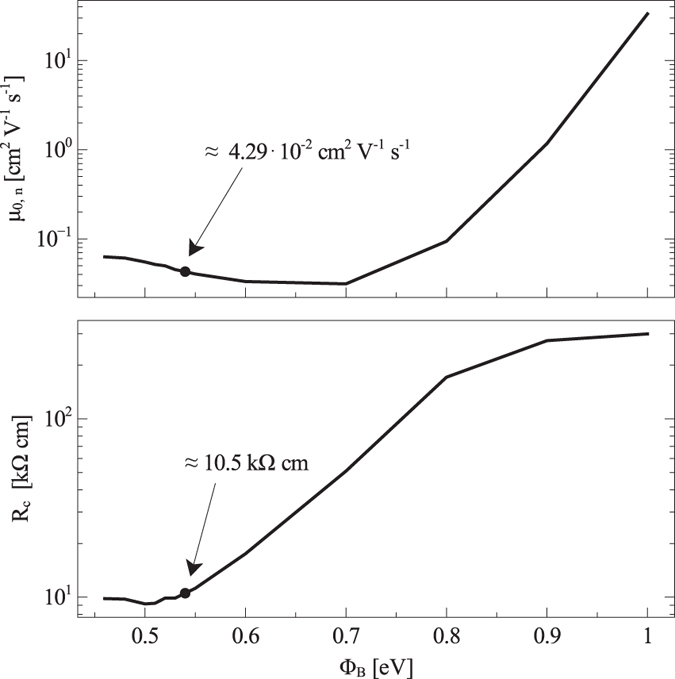
Low–field, low–density mobility *μ*_0,*n*_ (top) and contact resistance *R*_C_ (at *V*_g_ = +35 V, bottom), for different values of the injection barrier Φ_B_. The dots on the curves identify the Φ_B_ value that simultaneously yields in the best fitting of OTFT transfer characteristic curves and of MIS capacitor CF curves.

By fitting CV experimental data and assuming the nominal 1 eV barrier, we extract *σ* = 3.5 k_B_T (Fig. 2). We note that this value is slightly (about 15%) larger than what reported previously^4^. The difference can be ascribed to the more accurate modeling of the metal/semiconductor interface used in the present study, which accounts for the Schottky barrier lowering effect (see Section Boundary Conditions for the Drift–Diffusion Equations). But by fitting OTFT Current–Voltage (IV) characteristics (see Section Modeling the OTFT) we obtain ${\mu }_{\mathrm{0,}n}\simeq 33\,{{\rm{cm}}}^{{\rm{2}}}{{\rm{V}}}^{-{\rm{1}}}{{\rm{s}}}^{-{\rm{1}}}$ (Fig. 3), which is a completely unphysical value for P(NDI2OD-T2), whose low–field, low–density mobility should be around 10^−1^ ÷ 10^−2^ cm^2^ V^−1^ s^−1^. The large difference with respect to our previous work^4^ arises because the OTFT fitting takes into account the effect of *R*_C_ in the framework of the current crowding model^12, 13^. Also the contact resistance value extracted, in the range of hundreds of kΩ cm, is in contrast to the literature value of tens of kΩ cm for P(NDI2OD-T2) transistors with gold contacts^16^. With such a large barrier, contacts are poorly injecting: to compensate this phenomenon and to fit the experimental OTFT transfer characteristic curves the algorithm has to admit an exceedingly high value for *μ*_0,*n*_. Nonetheless, the transistor is severely contact limited and the fitting very bad (see Supplementary Information, Fig. SI7).

**Figure 4:**
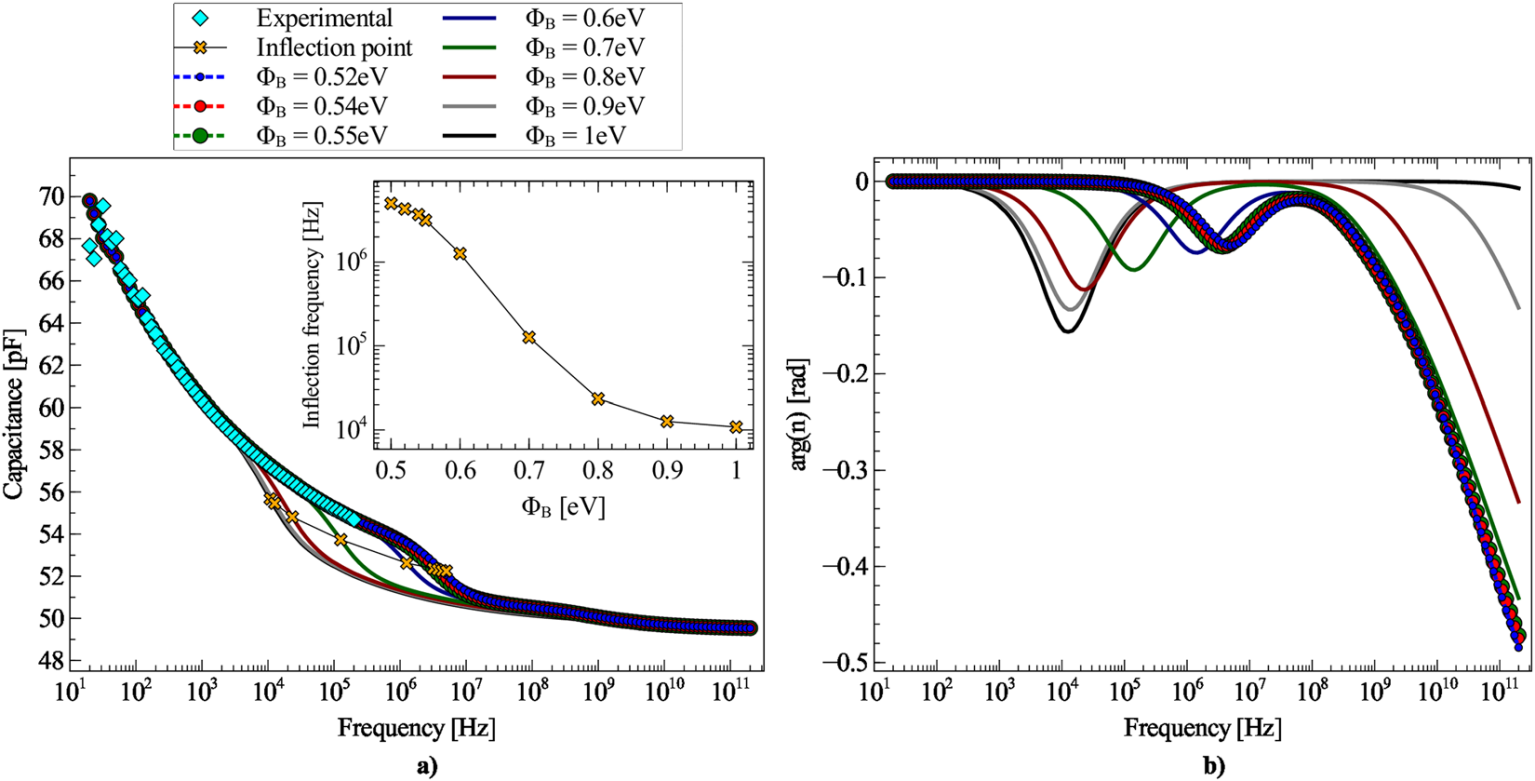
Results of Drift–Diffusion simulations of the MIS device in the high accumulation regime (V__g_= +35 V), for different values of the injection barrier Φ_B_. (**a**) CF curves. The inset shows the dependence of the inflection frequency on Φ_B_. Experimental CF characteristics shown for comparison (cyan diamonds). (**b**) Phase of the carrier density at the semiconductor/insulator interface ∠*n* (0).

The nominal barrier leads to results in contrast with experimental measurements also in the simulation of the CF curve of the MIS capacitor (see Section Post–processing Procedure for Producing CF Curves). Results are reported in Fig. 4a together with the experimental curve. The simulated curve shows a low frequency region and a high frequency region, separated by a transition region. In the former frequency region the MIS capacitor operates in the quasi–equilibrium regime; it does not appear as a plateau because with Poly(methyl methacrylate) (PMMA) dielectric *ε*_ins_ is frequency–dependent (in fact by simulating a CF curve with a constant *ε*_ins_ at low frequency a flat curve would be obtained, see Supplementary Information, Fig. SI3). A neater information can be gained by looking at the phase of the carrier density at the semiconductor/insulator interface, reported in Fig. 4b (its modulus is shown in Supplementary Information, Fig. SI2): at low frequency the phase is 0, implying that the accumulated channel is able to follow the modulation imposed by the small sinusoidal signal on the gate; but when the frequency rises, a local minimum occurs in ∠*n*(0), followed by a strong decrease, indicating that the channel lags behind the sinusoidal modulation.

We choose the CF curve inflection point as the demarcation frequency between the quasi–equilibrium and the out–of–equilibrium regions (see inset of Fig. 4a). With a barrier of 1 eV the simulated curve does not reproduce the experimental one: the former has an inflection point of about 10 kHz, the latter an inflection point that, albeit not clearly resolved in the measurement, is for sure in excess of 100 kHz.

It is consequently clear that the nominal barrier does not produce consistent results either in terms of OTFT contact resistance or in terms of MIS capacitor CF curves, and that the real Au/P(NDI2OD-T2) interface has to be indeed more effective in injecting charge carriers.

We then tried to reduce Φ_B_. For each value of Φ_B_, a value of *σ* is extracted by fitting CV curves; related values for *μ*_0,*n*_ and for the *R*_C_ are obtained from OTFT measurements and a new CF curve is simulated. The extracted DOS width is an increasing function of Φ_B_. In fact, the smaller the barrier, the larger the concentration of carriers at the metal/semiconductor interface. The population of thermal carriers close to the metal/semiconductor interface interferes with the gate attraction of charge close to semiconductor/insulator interface, thus making the CV curve less steep. This effect is sizable because the semiconductor film is relatively thin. Therefore, in order to fit experimental data, the smaller Φ_B_, the smaller σ.

As expected, the reduction of Φ_B_ fixes the aforementioned problems. The low–field, low–density carrier mobility is an increasing function of Φ_B_ and lies in the correct range (${\mu }_{\mathrm{0,}n}\simeq {10}^{-1}\div{10}^{-2}\,{{\rm{cm}}}^{{\rm{2}}}{{\rm{V}}}^{-{\rm{1}}}{{\rm{s}}}^{-{\rm{1}}}$) for Φ_B_ < 0.8 eV. The contact resistance *R*_C_ is an increasing function of Φ_B_ (see Fig. 3 and Supplementary Information, Fig. SI1), and for barriers lower than about 0.6–0.7 eV it lies in the expected range of tens of kΩ cm. *R*_C_ becomes independent of Φ_B_ for Φ_B_ < 0.55 eV, since it starts to be dominated and limited by carrier mobility rather than by carrier injection. As to CF curves, the inflection frequency is a decreasing function of Φ_B_: its dependence on Φ_B_ is correlated with the dependence of *R*_C_ on Φ_B_, meaning that the capacitor is contact limited rather than transport limited. A good agreement with experimental data is obtained for barriers smaller than about 0.6 eV.

Unfortunately by looking at the dependence of CV and CF curves on Φ_B_, only an upper bound for Φ_B_ can be found, but an optimum value for the barrier cannot be identified. Firstly, the various CV curves are all of comparable quality. In fact, the fitting procedure minimizes the distance between the peaks of measured and simulated d*C*/d*V* curves. Irrespective of Φ_B_, fittings are very good: peak distances (see Supplementary Information, Fig. SI4) are comparable for all the barrier values (being almost 3 orders of magnitude smaller than the peak heights themselves) and a clear minimum is not present. Secondly, the relative error between simulated and experimental CF curves is an increasing function of Φ_B_ but saturates for Φ_B_ < 0.6 eV (see Supplementary Information, Fig. SI5). This occurs because CF curves tend to become independent of Φ_B_ for Φ_B_ < 0.55 eV and the inflection frequency tends to saturate at about 5 MHz.

The situation is further complicated by the fact that in the very range of barrier values which is giving the best agreement between experimental and simulated CF curves and the most plausible value for *R*_C_, viz. Φ_B_ < 0.6 eV, the dependence of the DOS width on the barrier becomes very steep and hence the uncertainty on *σ* very large.

**Figure 5:**
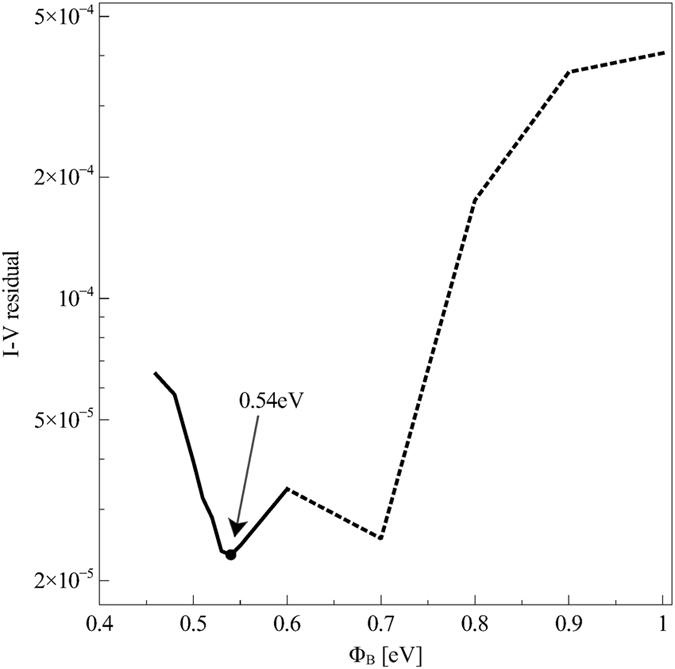
Residual of the least–squares fit of the OTFT transfer characteristic curves with contact resistance effects taken into account, at different values of the injection barrier Φ_B_. The solid line represents the range of barrier values that correspond to acceptable CF curves, as shown in Fig. 4a, while the dotted line is used for the range of barrier height values for which it is not possible to fit experimental CF curves and thus are to be considered unacceptable.

To identify the optimal barrier value we take advantage of OTFT transfer characteristic curves. These latter are sensitive to the DOS disorder degree through the dependence of the mobility on the carrier density: the higher is *σ*, the stronger the dependence of *μ* on the carrier density, the higher the (positive) curvature of transfer characteristic curves. If we look at the fit residuals of the experimental transfer characteristic curves, reported in Fig. 5, it can be appreciated that two relative minima exist: one for Φ_B_ = 0.7 eV and one for Φ_B_ = 0.54 eV. The former can be excluded because for a barrier of 0.7 eV the simulated CF curve does not reproduce the experimental one, while the latter value lies in the region where CF curves fit the experimental curve and where *μ*_0,*n*_ and *R*_C_ values are in agreement with the literature. The DOS width turns out to be 2.6 k_B_T and ${\mu }_{\mathrm{0,}n}\simeq 4\times {10}^{-2}\,{{\rm{cm}}}^{{\rm{2}}}{{\rm{V}}}^{-{\rm{1}}}{{\rm{s}}}^{-{\rm{1}}}$.

With respect to our former assessment^4^, the DOS width is slightly (about 15%) reduced and *μ*_0,*n*_ 30% larger.

The optimum value for Φ_B_ of 0.54 eV is sizeably smaller than the nominal barrier which assumes for gold a work function of 5.0 eV. But this latter value indeed refers to an atomically clean gold surface. In our case, the gold contact is solvent cleaned and processed in ambient air. As a consequence hydrocarbons are adsorbed on the gold surface, and reduce the metal surface dipole (which significantly contributes to the work function) by means of the so called push–back effect. It has been demonstrated that in polymer/gold interfaces gold behaves as if it had an *effective* work function of 4.5 eV^8^. With a P(NDI2OD-T2) LUMO level of 4.0 eV and considering such effective gold work function, the value for Φ_B_ of 0.54 eV we extract is perfectly conceivable.

## Conclusions

Experimental CV curves of a MIS capacitor in quasi–equilibrium can be equally well fitted by a relatively large set of values for the DOS width *σ* and for the Schottky barrier Φ_B_. This occurs because a small barrier produces – to a certain extent – the same smoothing effect on the CV curve of a large DOS width. As a consequence, uncertainties on Φ_B_ result in uncertainties on determining *σ*.

But if the dynamic, out–of–equilibrium behavior of the MIS capacitor and the transfer characteristic curve in the linear regime of the OTFT are additionally considered, such uncertainty can be drastically reduced. Based on the above considerations we presented a step–by–step procedure to determine the values of the DOS width *σ*, of the injection barrier Φ_B_ and of the low–field, low–density mobility *μ*_0,*n*_. In the first step a coarsely spaced set of possible values for Φ_B_ is selected and for each of those values *σ* and *μ*_0,*n*_ are determined by fitting CV curves for a MIS device and the transfer characteristics of an OTFT device respectively. This first step is performed using the fitting procedure defined in ref. 4, but with a refined model for metal–semiconductor interfaces including field induced barrier lowering. Once the values of *σ* and *μ*_0,*n*_ corresponding to each assumed value of the barrier height Φ_B_ are known, the CF curves of the MIS device can be simulated. These latter simulations can be used to restrict the range of acceptable Φ_B_ values by eliminating those values for which the frequency response of the device is inconsistent with respect to experiments. The set of candidate values for Φ_B_ within this restricted range is refined and the best fitting value is selected based on the residual of the least–squares distance of experimental to numerical IV curves.

When applied to the prototypical n-type polymer P(NDI2OD-T2), our strategy results in a value for *σ* of 2.6 k_B_T, for *μ*_0,*n*_ of 4 × 10^−2^ cm^2^ V^−1^ s^−1^ and for Φ_B_ of 0.54 eV. In particular, this latter can be rationalized considering that the polymer LUMO lies at 4.0 eV and the gold work function, taking into account its contamination by ambient air hydrocarbons, lies at 4.5 eV. This barrier permits to correctly estimate the OTFT contact resistances and to nicely reproduce MIS capacitor CF curves.

We expect that a wide class of materials can be analyzed using the proposed method. Firstly, the involved experimental setup and electrical measurements are simple, requiring the characterization of the capacitance as a function of frequency in MIS structures and linear transfer characteristics in OTFTs. The requisite on the patterning of the semiconductor in MIS capacitors does not hamper the method applicability: indeed, in this work we have met this requirement through a subtractive, wet-based approach, but should the associated chemistry prove harmful for the semiconductor, other solutions could be devised, such as subtractive, dry etching (e.g. laser patterning) or additive deposition. Secondly, the fitting procedure relies on the description of the DOS as a single Gaussian and of carrier transport according to the analytical formulation given in the framework of the EGDM; in case the spatial correlations are shown to be significant, the closely–related Extended Correlated Disorder Model (ECDM)^17^ could be implemented as well. As to the DOS shape, it has been recognized that only a DOS steeper than the exponential can reproduce the experimentally–observed mobility independence on carrier density at low concentration^18^. Actually, the experimental critical concentration at which the mobility starts to show density–dependence can only be explained assuming a DOS shape very close to the Gaussian one^19^. In general terms, it is possible that a single energy scale cannot effectively describe the real DOS (due to the superposition of inhomogeneously broadened, electronically inequivalent molecular states)^18^, as we actually verified for a recently synthesized n-type polymer^20^ that required two Gaussians in order to satisfactorily fit CV curves. In such case, to our knowledge, neither the EGDM nor any other analytical model is able to accurately describe carrier transport. As to the EGDM, we implemented the compact, analytical form for the mobility that is the result of a 1D parametrization of a 3D numerical modeling based on the Master Equation approach. As a consequence, we inherit the limits of such parametrization: the disorder parameter *σ* cannot substantially exceed about 8 k_B_T^20^, so that highly disordered materials are excluded. In addition, the model accuracy diminishes at very high concentrations (above 0.1*N*_0_) and fields (above 2*σ*/q*a*, with *a* the inter–site distance)^21^, but this has no actual impact: the highest carrier concentration in MIS structures does not exceed a few 0.001*N*_0_, and longitudinal fields are in the range of a few thousandths of 2*σ*/q*a* in OTFTs biased in the linear regime. Thirdly, thanks to the proper modeling of the metal/semiconductor interface, there is no strict need for a perfectly ohmic contact as long as the width of the quasi–static frequency plateau of the MIS capacitor is sufficiently large to be easily measured and identified.

To summarize, the combined exploitation of MIS CV and CF curves and OTFT transfer characteristic curves, enables the simultaneous assessment of the width of the Gaussian DOS, of the carrier mobility and of the metal/semiconductor injection barrier. The possibility to extract these three quantities by means of simple electrical measurements is extremely valuable, especially considering that the assessment of injection barriers usually requires dedicated and non–trivial experimental setups. The presented approach can thus easily empower a more detailed knowledge of organic semiconductors and foster further fundamental studies.

## Methods

### Experimental

Experimental data have been taken from ref. 4 where MIS capacitors were developed by spin–coating P(NDI2OD-T2) upon a gold bottom contact. The semiconductor was patterned^22^ to suppress the spurious effect of lateral carrier spreading^23–25^. PMMA was then spin coated as insulator and Aluminum was evaporated as gate contact. MIS capacitors were measured by means of an Agilent E4980A Precision LCR Meter, applying to the gate an oscillation amplitude of 100 mV of variable frequency superimposed to a biasing constant voltage.

On the same substrate OTFTs were realized in a staggered, top–gate bottom–contact configuration with gold source and drain contacts and Al gate and a channel width and length of 10 mm and 10, 20, 40 *μ*m. Transfer characteristic curves were measured applying a drain–to–source voltage of 5 V by means of Agilent B1500A Semiconductor Parameter Analyzer.

### Models for Numerical Simulations

Charge transport in the considered devices is modeled, in transient regime, by the Drift–Diffusion (DD) model, which is described in Section The Transient Drift–Diffusion Model^26–28^.

Transient simulations are used to compute the voltage and frequency dependence of the small–signal capacitance of the MIS capacitor, according to the numerical methods presented in Section Post–Processing Procedure for Producing CF Curves. The DD model features that are more important for this study are: (*i*) the boundary condition representing charge injection through the Schottky barrier at the metal/semiconductor interface, and (*ii*) the dependence of the mobility and of the diffusion coefficient on the DOS width. While useful for computing the capacitance over a wide range of frequencies, the full DD model turns out to be of too high complexity and of insufficient numerical accuracy for efficiently fitting measured low–frequency CV curves. For this reason we derived a modified version of the Non–Linear Poisson (NLP) model, reported in Section The Stationary Non–Linear Poisson Model, which includes a more accurate description of the contact injection barrier with respect to the previous work^4^, and is therefore fully consistent with the zero–frequency limit of the complete DD model.

The latter extended NLP model naturally describes the effect of the deviation from Einstein’s relation but, as it is derived for the quasi–static regime, it does not require to model the mobility coefficient.

The models used for computing the transfer characteristics of the OTFT device are object of discussion in Section Modeling the OTFT.

#### The Transient Drift-Diffusion Model

The geometrical setting for our numerical model of the MIS, shown in Fig. 1, consists of a one–dimensional schematization of the device along normal direction *z* to the semiconductor/insulator interface^4^. In the following we will denote by Ω_sc_, Ω_ins_ the semiconductor and insulator regions respectively (such that Ω = Ω_sc_ ∪ Ω_ins_ is the whole computational domain) and by *T* the simulated timespan.

Under the assumption that characteristic lengths of the simulation domain are small compared to the electromagnetic field wavelength, the electrostatic potential *φ* may be related to the net charge density *ρ* via the Poisson equation

1 $$-\frac{{\rm{\partial }}}{{\rm{\partial }}z}(\varepsilon \frac{{\rm{\partial }}\phi }{{\rm{\partial }}z})-\rho =0,\quad {\rm{i}}{\rm{n}}\,{\rm{\Omega }}\times T,$$

where *ε* is the electrical permittivity and *ρ* the charge density. Assuming only electrons as charge carriers (having volume density *n*) and neglecting holes, trapped charges and dopant ion density, *ρ* = −q*n*, where q is the quantum of charge; subsequently, *ρ* = 0 in insulating regions. Charge conservation is expressed by the current continuity equation for electrons

2 $$\frac{{\rm{\partial }}n}{{\rm{\partial }}t}-\frac{1}{{\rm{q}}}\frac{{\rm{\partial }}{J}_{n}}{{\rm{\partial }}z}=0,\quad {\rm{i}}{\rm{n}}\,{{\rm{\Omega }}}_{{\rm{s}}{\rm{c}}}\times T,$$

where *J*_*n*_ the electron current density, which is assumed to consist of both drift and diffusion contributions:

3 $${J}_{n}={\rm{q}}({D}_{n}\frac{{\rm{\partial }}n}{{\rm{\partial }}z}-{\mu }_{n}n\frac{{\rm{\partial }}\phi }{{\rm{\partial }}z}),$$

*D*_*n*_ and *μ*_*n*_ denoting the diffusion and mobility coefficients respectively.

In the following we will assume a Gaussian shape for the DOS^4, 18, 20, 29^ which may be expressed as

4 $$N(E,{E}_{{\rm{L}}{\rm{U}}{\rm{M}}{\rm{O}}})=\displaystyle \frac{{N}_{0}}{\sqrt{2\pi {\sigma }^{2}}}\,\,\cdot\, \,\exp [-\frac{{(E-{E}_{{\rm{L}}{\rm{U}}{\rm{M}}{\rm{O}}})}^{2}}{2{\sigma }^{2}}],$$

where *N*_0_ denotes the total density of hopping sites, *E*_LUMO_ the LUMO energy level and *σ* the DOS width. Even though in our method the DOS could in principle be given by a superposition of multiple Gaussians, for the tested P(NDI2OD-T2) semiconductor we already proved that a single Gaussian yields a good agreement between simulations and experimental data^4^. Under this assumption for the DOS, it is possible to express the mobility coefficient according to the EGDM^21^:

5 $${\mu }_{n}={\mu }_{0,n}\,{g}_{1}(n)\,{g}_{2}({\mathcal{E}}),$$

where *μ*_0,*n*_ is the low–field and low–density mobility and the two enhancement factors *g*_1_ and *g*_2_ account respectively for the dependence on the carrier density *n* and on the electric field E. The analytical expression for the enhancement factors is presented in Section The EGDM Mobility Model.

Charge injection/extraction at the metal/semiconductor interface is modeled by imposing that carrier density at the contact relaxes with finite velocity *v*_*n*_ to an equilibrium value *n*_0_ which depends on the intensity and direction of the normal electric field at the contact.

$${J}_{n}={\rm{q}}\,{v}_{n}\cdot ({n}_{0}-n).$$

Following ref. 30 we adopt models for *v*_*n*_ and *n*_0_ that result in a variant of the well known injection model developed by Scott and Malliaras^26, 31^. Further details on the boundary conditions imposed on the DD system are collected in Section Boundary Conditions for the Drift–Diffusion Equations.

#### The EGDM Mobility Model

The EGDM enhancement factors *g*_1_, *g*_2_ in equation (5) are computed via the following relations^6, 32^:

$$\begin{array}{cc}{g}_{1}(n) & \,=\frac{{N}_{0}}{n}\exp [\frac{{E}_{{\rm{F}}}-{E}_{{\rm{L}}{\rm{U}}{\rm{M}}{\rm{O}}}}{{{\rm{k}}}_{{\rm{B}}}{\rm{T}}}+\frac{1}{2}{(\frac{\sigma }{{{\rm{k}}}_{{\rm{B}}}{\rm{T}}})}^{2}],\\ {g}_{2}({\mathcal{E}}) & \,=\exp \{0.44[{(\frac{\sigma }{{{\rm{k}}}_{{\rm{B}}}{\rm{T}}})}^{3/2}-2.2][\sqrt{1+0.8{(\frac{{\rm{q}}{\mathcal{E}}}{{N}_{0}^{1/3}\sigma })}^{2}}-1]\},\end{array}$$

The diffusivity and mobility coefficients *D*_*n*_ and *μ*_*n*_ in equation (3) are related via the generalized Einstein relation^21^:

$${D}_{n}={g}_{3}(n({E}_{{\rm{L}}{\rm{U}}{\rm{M}}{\rm{O}}},{E}_{{\rm{F}}}))\displaystyle \frac{{{\rm{k}}}_{{\rm{B}}}{\rm{T}}}{{\rm{q}}}{\mu }_{n}.$$

Assuming Fermi–Dirac statistics for the occupation probability of electron energy states, the electron density may be expressed as

6 $$n({E}_{{\rm{L}}{\rm{U}}{\rm{M}}{\rm{O}}},{E}_{{\rm{F}}})=\displaystyle \underset{-{\rm{\infty }}}{\overset{+{\rm{\infty }}}{\int }}N(E,{E}_{{\rm{L}}{\rm{U}}{\rm{M}}{\rm{O}}})\displaystyle \frac{1}{1+\exp (\displaystyle \frac{E-{E}_{{\rm{F}}}}{{{\rm{k}}}_{{\rm{B}}}{\rm{T}}})}{\rm{d}}E,$$

where *E*_F_ denotes the Fermi level, k_B_ the Boltzmann constant and T the temperature, and the dimensionless diffusion enhancement factor *g*_3_ is given by

$${g}_{3}(n)={({{\rm{k}}}_{{\rm{B}}}{\rm{T}}\,\displaystyle \frac{{\rm{\partial }}n}{{\rm{\partial }}{E}_{{\rm{F}}}})}^{-1}\,n.$$

The partial derivative ∂*n*/∂*E*_F_ can be easily computed by substituting equations (4) into (6) and by applying a suitable change of variables^4^ for the sake of convenience, yielding:

$$n({E}_{{\rm{L}}{\rm{U}}{\rm{M}}{\rm{O}}},{E}_{{\rm{F}}})=\displaystyle \frac{{N}_{0}}{\sqrt{\pi }}\displaystyle \underset{-{\rm{\infty }}}{\overset{+{\rm{\infty }}}{\int }}{e}^{-{\eta }^{2}}{[1+\exp (\displaystyle \frac{\sqrt{2}\sigma \eta +{E}_{{\rm{L}}{\rm{U}}{\rm{M}}{\rm{O}}}-{E}_{{\rm{F}}}}{{{\rm{k}}}_{{\rm{B}}}{\rm{T}}})]}^{-1}{\rm{d}}\eta .$$

#### Boundary Conditions for the Drift-Diffusion Equations

In the transient simulation, we account for parasitic contributions (due to coupling between the top metal gate and the bottom metal line running to the bonding pad, fringing electric field lines running from the perimeter of the bottom metal to the top metal) by assuming that a capacitor *C*_sb_ is connected in parallel to the 1D MIS device^4^. Let now *z*_sc_ and *z*_ins_ be the thickness of the semiconductor and insulator layer respectively (so that Ω_sc_ = {*z*: − *z*_sc_ ≤ *z* ≤ 0} and Ω_ins_ = {*z*: 0 ≤ *z* ≤ *z*_ins_}). The values of the electric potential at the ends of the computational domain are given by

$$\{\begin{array}{ccc}{\phi |}_{z=-{z}_{{\rm{s}}{\rm{c}}}} & = & -{{\rm{\Phi }}}_{{\rm{B}}}/{\rm{q}}\\ {\phi |}_{z={z}_{{\rm{i}}{\rm{n}}{\rm{s}}}} & = & {V}_{{\rm{g}}}+{V}_{{\rm{s}}{\rm{h}}{\rm{i}}{\rm{f}}{\rm{t}}},\end{array}$$

where Φ_B_ is the zero–field value of the Schottky barrier height and *V*_shift_ is a model parameter accounting for effects such as permanent dipoles, fixed charge in dielectrics or metal work function mismatch^4^.

At the metal/semiconductor interface we model charge injection/extraction phenomena by imposing the following Robin boundary condition on the continuity equation:

7 $${{J}_{n}|}_{z=-{z}_{{\rm{s}}{\rm{c}}}}={\rm{q}}\,{v}_{n}\cdot ({n}_{0}-{n|}_{z=-{z}_{{\rm{s}}{\rm{c}}}}),$$

where *n*_0_ is the equilibrium charge density, expressed as

$${n}_{0}=\displaystyle \frac{{N}_{0}}{\sqrt{\pi }}\displaystyle \underset{-{\rm{\infty }}}{\overset{+{\rm{\infty }}}{\int }}{e}^{-{\eta }^{2}}{[1+\exp (\displaystyle \frac{\sqrt{2}\sigma \eta +{{\rm{\Phi }}}_{{\rm{B}}}}{{{\rm{k}}}_{{\rm{B}}}{\rm{T}}}-{\rm{\Delta }})]}^{-1}{\rm{d}}\eta .$$

Following the description in ref. 26, the coefficient *Δ* accounts for Schottky barrier lowering/increase depending on the field at the electrode:

$${\rm{\Delta }}=\{\begin{array}{c}3\sqrt{f},\quad {\textstyle \text{if}}\,f\ge 0\,({\textstyle \text{carrier injection}})\\ f/4,\quad {\textstyle \text{if}}\,f < 0\,({\textstyle \text{carrier extraction}}),\end{array}$$

where $f={\rm{q}} {\mathcal E} {r}_{{\rm{c}}}/({{\rm{k}}}_{{\rm{B}}}{\rm{T}})$ is the reduced electric field and *r*_c_ = q^2^/(4*πε*_sc_k_B_T) the Coulomb radius. The recombination velocity *v*_*n*_ in the injection regime is given by

$${v}_{n}(f)=\displaystyle \frac{4\pi \varepsilon {({{\rm{k}}}_{{\rm{B}}}{\rm{T}})}^{2}{\mu }_{0,n}}{{{\rm{q}}}^{3}}(\frac{1}{{\psi }^{2}(f)}-f),\quad \psi (f)={f}^{-1}+{f}^{-\frac{1}{2}}-{f}^{-1}{(1+2{f}^{\frac{1}{2}})}^{\frac{1}{2}},$$

while under the carrier extraction it is cut off at its zero–field value, viz. *v*_*n*_(*f*) = *v*_*n*_(0) if *f* < 0. Finally, as leakage currents through the gate insulator are neglected, at the semiconductor/insulator interface a homogeneous Neumann condition holds:

8 $${{J}_{n}|}_{z=0}=0.$$

#### The Stationary Non–Linear Poisson Model

The DD equations presented completely describe the devices under study and could in principle be used in any operation regime (stationary, transient, AC,…). Unfortunately, given the strict tolerances and the large number of simulation runs required by the parameter fitting algorithm described in ref. 4, the DD may lead to unaffordable computational costs. Fortunately, this limitation can be overcome in the simulation of CV curves in the static regime, as the model complexity can be significantly reduced. In the stationary regime, equation (8) implies that *J*_*n*_ = 0 everywhere and that the carrier density does not depend on time. As a result the Fermi potential *E*_F_ is constant in both space and time (and may eventually be set to 0 for simplicity). Therefore the system (1), (2) reduces to the following NLP equation:

$$\{\begin{array}{c}-\displaystyle \frac{{\rm{\partial }}}{{\rm{\partial }}z}(\varepsilon \displaystyle \frac{{\rm{\partial }}\phi }{{\rm{\partial }}z})+{\rm{q}}\,n(\phi )=0,\quad {\textstyle \text{in}}\,{\rm{\Omega }}\\ n(\phi )=\displaystyle \frac{{N}_{0}}{\sqrt{\pi }}\displaystyle \underset{-{\rm{\infty }}}{\overset{+{\rm{\infty }}}{\int }}{e}^{-{\eta }^{2}}{[1+\exp (\displaystyle \frac{\sqrt{2}\sigma \eta -{\rm{q}}\phi }{{{\rm{k}}}_{{\rm{B}}}{\rm{T}}}-{\rm{\Delta }})]}^{-1}{\rm{d}}\eta \\ {\phi |}_{z=-{z}_{{\rm{s}}{\rm{c}}}}=-{{\rm{\Phi }}}_{{\rm{B}}}/{\rm{q}}\\ {\phi |}_{z=-{z}_{{\rm{s}}{\rm{c}}}}={V}_{{\rm{g}}}+{V}_{{\rm{s}}{\rm{h}}{\rm{i}}{\rm{f}}{\rm{t}}}.\end{array}$$

We finally remark that, unlike the model presented in ref. 4, *φ* does no longer represent the potential associated to the LUMO level due to the barrier lowering *Δ*:

$${E}_{{\rm{LUMO}}}(z)-{E}_{{\rm{F}}}=-{\rm{q}}\phi (z)-{{\rm{k}}}_{{\rm{B}}}{\rm{T}}{\Delta },$$

and that the boundary condition at the metal/semiconductor interface is consistent with equation (7), i.e. ${n|}_{z=-{z}_{{\rm{s}}{\rm{c}}}}={n}_{0}$. The procedure for accurately deducing the device low–frequency capacitance from charge and potential profiles is described in refs^4, 33^.

#### Modeling the OTFT

Once the DOS width *σ* has been extracted by fitting static CV curves, the low–field, low–density mobility *μ*_0,*n*_ can be determined by computing OTFT transfer characteristics in the linear regime. The drain–to–source current is expressed as

$${I}_{{\rm{DS}}}({V}_{{\rm{g}}})=\frac{{V}_{{\rm{DS}}}}{{R}_{{\rm{tot}}}({V}_{{\rm{g}}})},$$

where *V*_DS_ is the potential drop across the channel and *R*_tot_ is the total device resistance, accounting for both the channel and the contact resistance contributions:

$${R}_{{\rm{tot}}}={R}_{{\rm{ch}}}+{R}_{{\rm{C}}},$$

where the channel resistance *R*_ch_ is given by:

9 $${R}_{{\rm{c}}{\rm{h}}}={[\displaystyle \frac{W}{L}\displaystyle \underset{-{z}_{{\rm{s}}{\rm{c}}}}{\overset{0}{\int }}{\rm{q}}{\mu }_{n}(z)n(z){\rm{d}}z]}^{-1},$$

*W* and *L* being the channel width and length respectively. As for *R*_C_, we followed the physical description provided in refs^12, 13^, in the framework of current crowding regime. By considering the contributions due to the current flow across the accumulated channel and along the semiconductor layer, characterized by a sheet resistance *R*_sh_ and a resistance per unit area *R*_y_ respectively, the contact resistance is computed as:

$${R}_{{\rm{C}}}=\frac{{R}_{{\rm{y}}}}{W{L}_{0}\,\tanh ({L}_{{\rm{ov}}}/{L}_{0})},$$

where *L*_ov_ is the overlap length between the gate and the source/drain electrodes, *L*_0_ = (*R*_y_/*R*_sh_)^1/2^ and

10 $${R}_{{\rm{s}}{\rm{h}}}={[\underset{-{z}_{{\rm{s}}{\rm{c}}}}{\overset{0}{\int }}{\rm{q}}{\mu }_{n}(z)n(z){\rm{d}}z]}^{-1},$$

11 $${R}_{{\rm{y}}}=\underset{-{z}_{{\rm{s}}{\rm{c}}}}{\overset{0}{\int }}{[{\rm{q}}{\mu }_{n}(z)n(z)]}^{-1}{\rm{d}}z.$$

**Figure 6:**
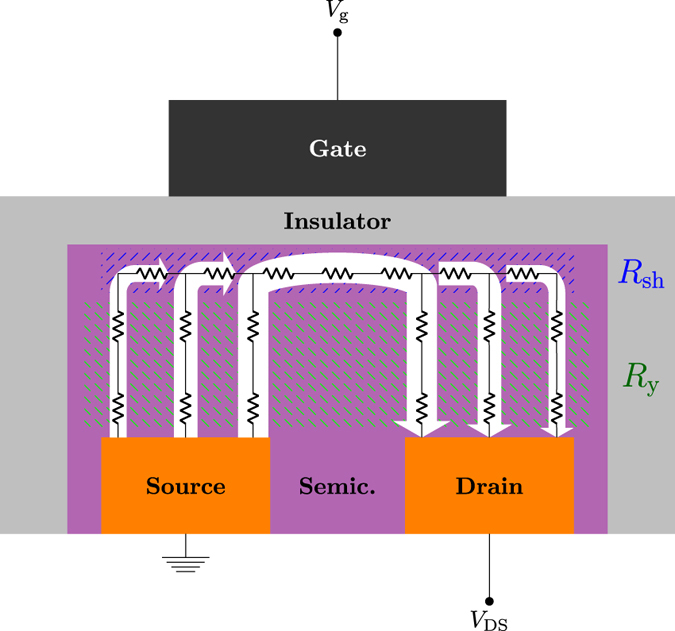
Sketch of the cross–section of the OTFT under current crowding.

The integrand functions in equations (9), (10) and (11) are computed by simulating, as highlighted in Fig. 6, two different one–dimensional cross–sections along the *z* direction corresponding to the middle of the channel (*R*_ch_, *R*_sh_) and to the source contact (*R*_y_) respectively.

The mobility coefficient *μ*_*n*_(*z*) is expressed through the EGDM model (5), where the enhancement factors *g*_1_(*n*) and *g*_2_(E) (E = *V*_DS_/*L* is the drain–to–source field) can be easily computed once *σ* is known, as described in Section The EGDM Mobility Model. Therefore, the total current *I*_DS_ is known up to the multiplicative constant *μ*_0,*n*_, which is then extracted by fitting numerical and experimental IV curves through a least–squares procedure. The fitting residual has been exploited to determine the optimal value of Φ_B_, as shown in Fig. 5.

#### Post–processing Procedure for Producing CF Curves

The DD system has been employed to simulate AC regimes over a timespan $T=\{t:{t}_{\min }\le t\le {t}_{\max }\}$ (where ${t}_{\min }\le \mathrm{0,}\,{t}_{\max }\ge 0$ are assumed for the sake of simplicity). In order to compute CF curves numerically, we apply gate voltages of the form:

12 $${V}_{{\rm{g}}}(t)=\{\begin{array}{cc}\bar{V}, & t\in [{t}_{min},\,0]\\ \bar{V}+{V}_{0}\,\sin (\omega t), & t\in [0,\,{t}_{max}],\end{array}$$

where $\bar{V}$ is the selected bias (attained through a ramp signal) and *ω* is the angular frequency of the sinusoid; its amplitude *V*_0_ is set equal to 0.1 V, so that results are comparable to experimental measurements. We simulate 5 periods of oscillation, as a compromise between accuracy and computational cost, by choosing *t*_max_ = 10*π*/*ω*.

The computation of the equivalent capacitance follows from a Fourier analysis, assuming that the simulated current *I* flowing from the generator *V*_g_ is due to a parallel RC circuit with resistance *R*_*p*_ and capacitance *C*_*p*_. The total current of the equivalent circuit is

$$I=\frac{{V}_{0}}{{R}_{p}}\,\sin (\omega t)+{V}_{0}\omega {C}_{p}\,\cos (\omega t),$$

which provides

$${R}_{p}=\frac{{V}_{0}}{\alpha },\quad {C}_{p}=\frac{\beta }{{V}_{0}\omega },$$

where

$$\alpha =\displaystyle \frac{\underset{0}{\overset{{t}_{max}}{\int }}I\,\sin (\omega t){\rm{d}}t}{\underset{0}{\overset{{t}_{max}}{\int }}\,\sin \,{(\omega t)}^{2}\,{\rm{d}}t},\quad \beta =\displaystyle \frac{\underset{0}{\overset{{t}_{max}}{\int }}I\,\cos (\omega t)\,{\rm{d}}t}{\underset{0}{\overset{{t}_{max}}{\int }}\,\cos \,{(\omega t)}^{2}\,{\rm{d}}t}.$$

Then the equivalent capacitance *C*_*p*_ is easily computed once the numerical simulation has provided the total current *I*.

## Electronic supplementary material

Supplementary Information
