# Supplementary material for: Simultaneous Extraction of Density of States Width, Carrier Mobility and Injection Barriers in Organic Semiconductors
Source: Sci Rep. 2017 Jun 19;7:3803. doi: 10.1038/s41598-017-03882-8 (PMC5476661; doi:10.1038/s41598-017-03882-8)
Supplement: Supplementary file 1 — Supplementary Information [file 41598_2017_3882_MOESM1_ESM.pdf]

Supplementary Information

Simultaneous Extraction of Density of States Width, Carrier Mobility and Injection Barriers in Organic Semiconductors

Pasquale Claudio Africa, Carlo de Falco, Francesco Maddalena, Mario Caironi, Dario Natali

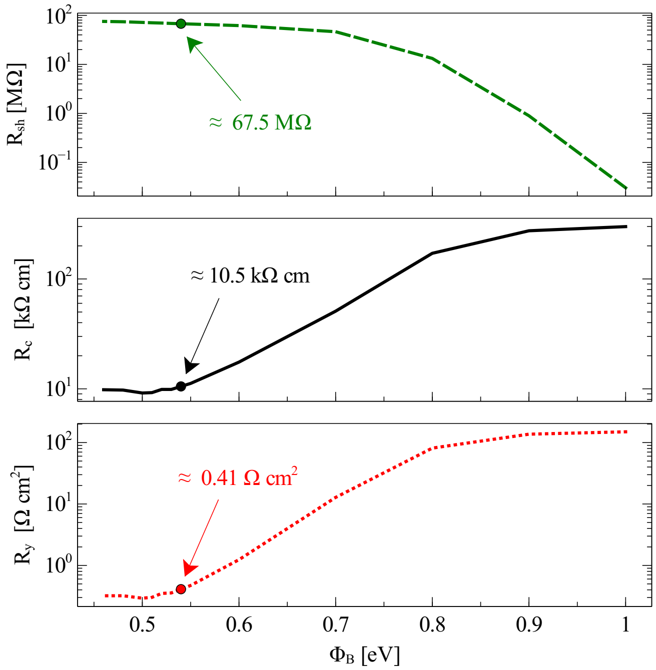

Figure SI1. Sheet resistance R_{sh}, access resistance R_y and the resulting contact resistance R_c, extracted at $V_g = +35\,V$ for different values of the injection barrier Φ_B. The dots on the curves identifies the Φ_B value which simultaneously yields in the best fitting of OTFT transfer characteristic curves and of MIS capacitor CF curves.

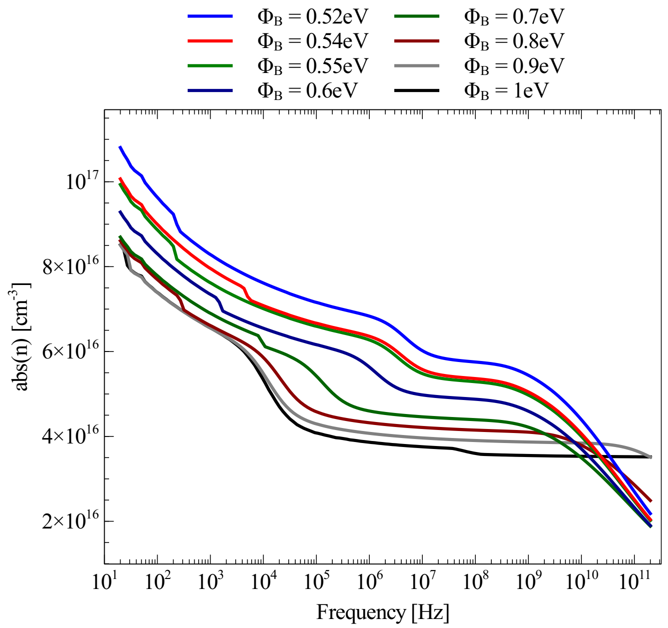

Figure SI2. Modulus of the carrier density at the semiconductor/insulator interface $|n(0)|$ in the high accumulation regime ($V_g = +35\,V$).

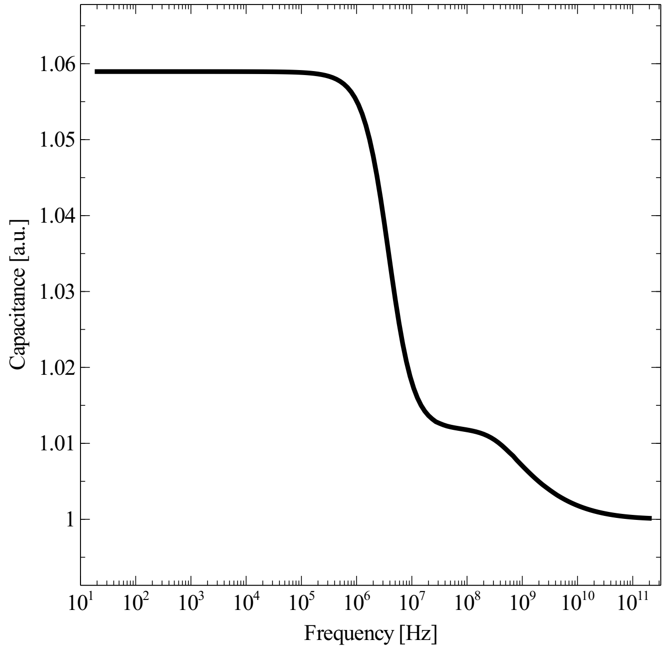

Figure SI3. Capacitance–Frequency characteristic at the optimal barrier $\Phi_B = 0.54\,eV$, simulated with a constant insulator permittivity ε_{ins}.

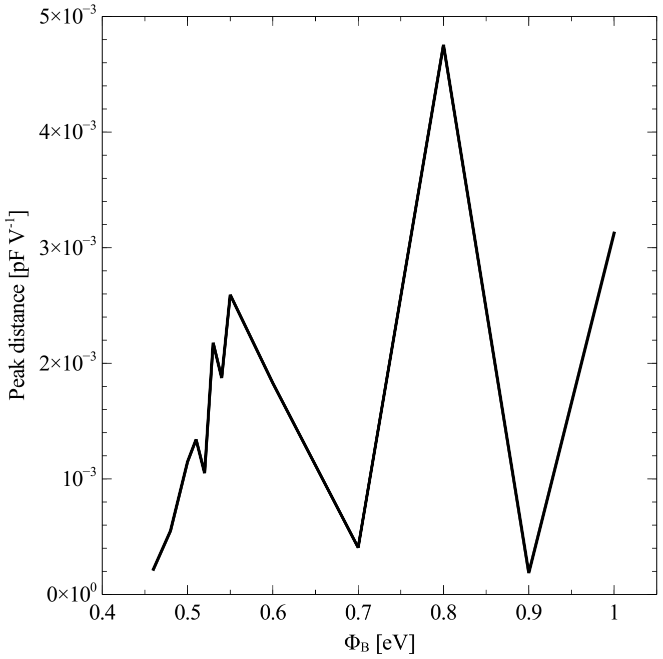

Figure SI4. Distance between the peaks of measured and simulated $\mathrm{d}C/\mathrm{d}V$ curves at different values of the injection barrier Φ_B.

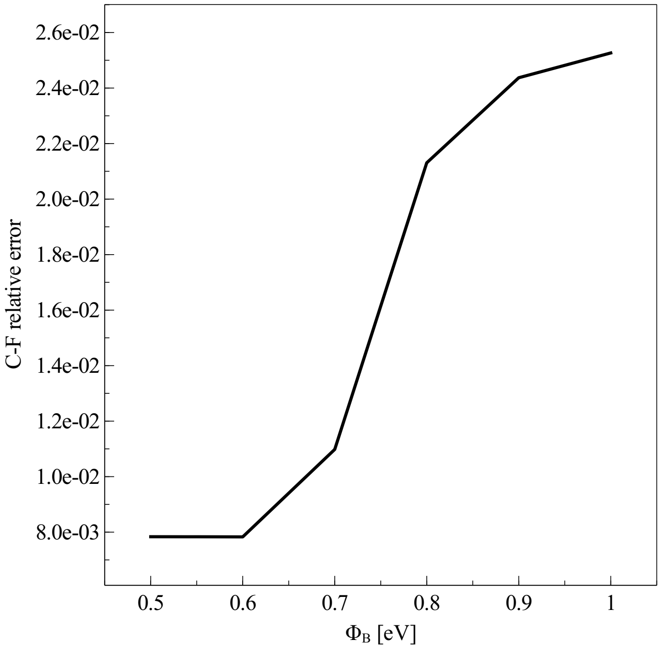

Figure SI5. Relative error between experimental and simulated Capacitance–Frequency characteristics for each barrier value Φ_B.

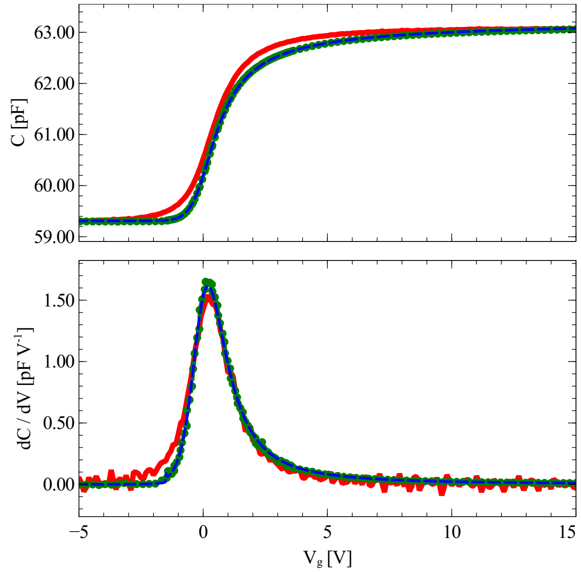

(a) 233Hz.

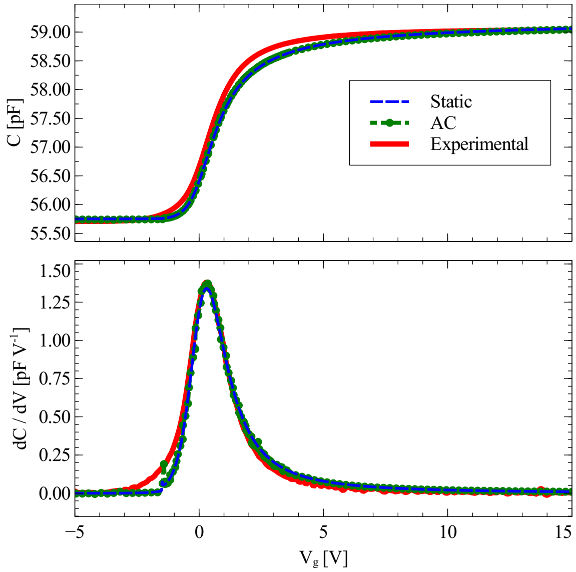

(b) 2332Hz.

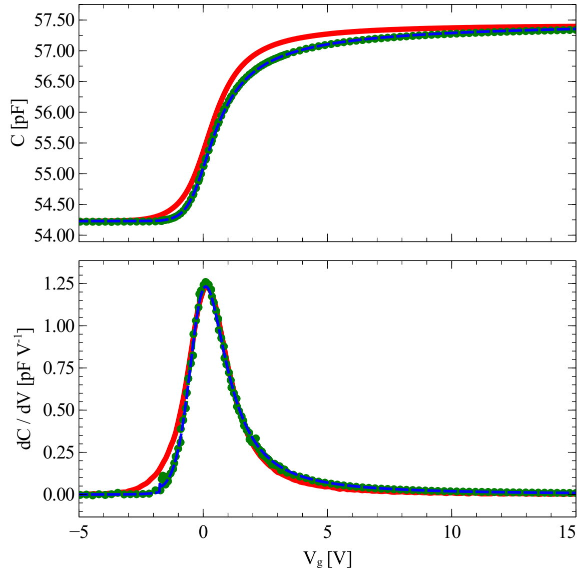

(c) 9283Hz.

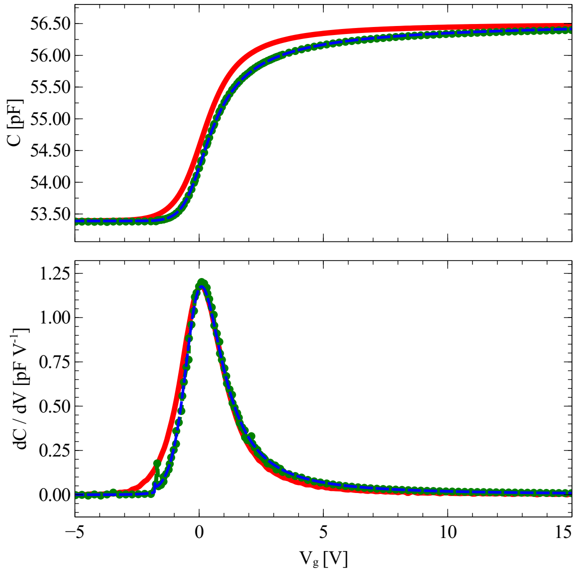

(d) 23318Hz.

Figure SI6. Capacitance–Voltage characteristics computed at different frequencies using the extracted optimum values of σ, Φ_B and μ_0. A perfect agreement is noticeable between the static simulation, performed using the Non–Linear Poisson model, and the transient simulation, performed using the complete Drift–Diffusion model in AC regime. Firstly, the CV curve at frequency 9283 Hz has been exploited for the fitting algorithm, then the same set of parameters has been used to simulate the frequencies 233 Hz, 2332 Hz and 23318 Hz, showing a good match to experimental data and thus the physical meaningfulness of the parameters extracted.

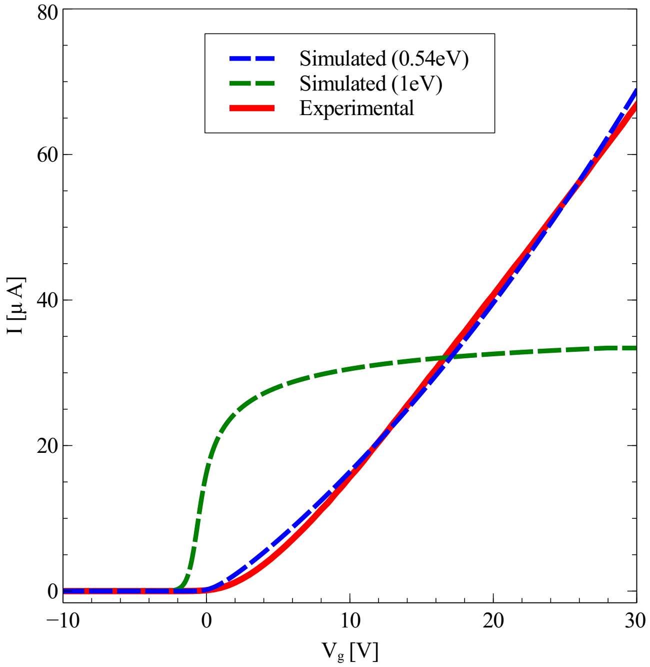

Figure SI7. Comparison between experimental (red) and simulated OTFT transcharacteristics. Simulated curves are shown for the nominal barrier value $\Phi_B = 1\,\text{eV}$ (green) and the extracted optimum $\Phi_B = 0.54\,\text{eV}$ (blue). The simulation with the nominal barrier results in a severely contact-limited transistor, in fact the transfer characteristic curve becomes flat for high values of V_g.
